# Urgent EMS managed out-of-hospital delivery dispatches in Helsinki

**DOI:** 10.1186/s13049-016-0285-5

**Published:** 2016-07-25

**Authors:** Jussi Pirneskoski, Katja Peräjoki, Mika Nuutila, Markku Kuisma

**Affiliations:** 1 Department of Emergency Medicine and Services, Helsinki University and Helsinki University Hospital, Haartmaninkatu 4, Helsinki, PL 340, 00290 HUS Finland; 2 Department of Obstetrics and Gynaecology, Helsinki University and Helsinki University Hospital, Haartmaninkatu 2, Helsinki, PL 140, 00290 HUS Finland

**Keywords:** Emergency medical services, Out-of-hospital delivery, Out of hospital birth, Prehospital delivery, Birth before arrival, Unplanned delivery, EMS

## Abstract

**Background:**

The aim of this study was to examine Helsinki Emergency Medical Services (EMS) and hospital records to determine the incidence and possible complications of out-of-hospital deliveries managed by EMS in Helsinki.

**Methods:**

We retrospectively analysed all urgent ambulance dispatches relating to childbirth in Helsinki from January 1, 2010 to December 31, 2014 with further analysis of hospital records for the out-of-hospital deliveries. Patients were divided in to two groups: those who delivered before reaching hospital and those who did not deliver before reaching hospital and differences between groups were analysed. Deliveries with gestational age of at least 22 + 0 weeks were considered as births in statistical analysis as this is the current national practice.

**Results:**

There were 799 urgent dispatches during the study period. In 102 (12.8 %) of these delivery took place before reaching the hospital. The incidence of EMS managed out-of-hospital delivery was found to be 3.0/1000 births. The annual number of out-of-hospital deliveries attended by EMS increased from 15 in 2010 to 28 in 2014. No stillbirths were reported. Neither maternal or perinatal deaths nor major maternal complications were noted in the study population.

**Discussion:**

Out-of-hospital deliveries represent a small minority of EMS calls and remain a challenge to maintaining professional capabilities. Small sample size might have limited the ability of the study to pick up rare complications.

**Conclusions:**

The amount of out-of-hospital deliveries in Helsinki increased during the five-year study period. There were no maternal or perinatal mortality or major complications resulting in long-term sequelae associated with the EMS-managed out-of-hospital births.

## Background

The current evidence suggests that especially unplanned out-of-hospital deliveries are associated with increased perinatal mortality and morbidity [1, 2]. The recommendations regarding planned home birth vary in different countries [3, 4], in Finland the current standard is delivery at hospital and home births are not encouraged [5]. Centralisation of deliveries to larger units has decreased the amount of delivery hospitals in Finland by a third in the last 25 years [6]. Similar trend has been seen in Norway and has led to an increase in the number of out-of-hospital deliveries in both countries [1, 6]. The delivery hospital network is currently under review and even further reduction in the number of delivery hospitals is planned.

Helsinki’s out-of-hospital emergency medical services (EMS) are covered by Helsinki EMS. The general view amongst the physicians working for Helsinki EMS during the last years has been that out-of-hospital deliveries are on the rise even though the number of delivery units in Helsinki area has remained constant. This prompted a review of ambulance records to find out whether the number of out-of-hospital deliveries were indeed increasing and what were the outcomes for the mothers and the infants.

The aim of this study was to examine the ambulance records to find out the number of calls relating to childbirth and the number of out-of-hospital deliveries. Secondarily, should the number of out-of-hospital deliveries be on the rise, to examine the hospital records and try to establish causal factors and to review possible complications during the out-of-hospital deliveries.

## Methods

### Study setting and type

This retrospective cohort study was conducted in Helsinki EMS. The Operative Division of Helsinki University Hospital approved the study plan. The approval included access to patients’ medical records. No informed consent was required.

### Study location and maternity hospitals

Helsinki is the capital of Finland with approximately 620,000 inhabitants and area of 715 km^2^. There are two delivery hospitals in Helsinki which both are part of the Helsinki University Hospital. About 6800 mothers living in Helsinki give birth in these two hospitals every year. Both hospitals are within 30 km from any inhabited area of Helsinki with majority of the population living even closer to the hospitals.

### Dispatching and EMS

Dispatching service in Finland is run by national Emergency Response Centre Agency. The dispatchers work in five different centres dispatching the units of a number of EMS systems working in the dispatching centre area. The emergency dispatches are categorised from A to D, A being most urgent. Dispatch categories A and B are used when immediate risk to basic vital functions is either apparent or suspected by the dispatcher.

Helsinki EMS is three tiered and is operated by Helsinki City Rescue Department and responds to around 55,000 calls yearly. The first tier consists of basic life support (BLS) ambulances manned by emergency medical technicians (EMTs). The second tier consists of advanced life support (ALS) ambulances manned with paramedics and an on-duty medical supervisor unit. A physician-staffed mobile intensive care unit (MICU) makes up the third tier. In addition to attending life-threatening calls, the MICU physician supports all other units with teleconsultation services. All units are equipped with electronic patient reporting system and online data transmission. Midwives are not a part of the EMS system and the units described above handle all calls including the ones relating to childbirth.

### Data collection

We included data from all ambulance dispatches relating to childbirth in the A and B dispatch categories in Helsinki area from January 1, 2010 to December 31, 2014. Dispatching data was collected from Helsinki EMS call and patient records. Out-of-hospital birth was determined as a delivery of a child of at least 22 weeks of gestational age at any point of time before the mother reached the hospital. Births before 22 + 0 weeks were considered as spontaneous abortions and not included in the birth statistics.

Regarding the dispatching and EMS efficiency, we recorded the time of call, the time of first unit dispatched and the time of the first unit reaching the patient. In addition, the presence of the physician-staffed MICU on scene was noted. From the ambulance records we also extracted data regarding whether the delivery took place before the unit had arrived on scene, when the unit was on scene or if the delivery did not take place before reaching the hospital. Apgar scores given by the ambulance staff were collected for babies born before reaching the hospital. We also extracted the basic maternal haemodynamic measurements (pulse rate, blood pressure) and the reason for prehospital delivery from the ambulance records.

From hospital records we extracted the date of birth, gestational age, gravidity and parity of the patient. We also acquired the nationality and native language of the patients. Hospital records also included an estimation of the total amount of maternal blood loss during the delivery. The delivery complications for the mothers who had given birth before reaching hospital and the children born out-of-hospital as stated by the hospital records were collected. For the children that were born out-of-hospital weight, height and head circumference of the child measured in hospital were recorded along with the sex of the child.

For comparison we acquired the Helsinki birth statistics from the National Institute for Health and Welfare, which keeps a national birth registry of all births in Finland based on information required to be submitted by the hospitals.

### Statistical analysis

For statistical analysis the patients were classified in to two groups: those who delivered before reaching hospital (Group 1) and those whose delivery did not take place before reaching hospital (Group 2). We analysed differences between the groups using Mann–Whitney *U*-test for the applicable variables. Statistical analysis was performed using GraphPad Prism 6.0 g (GraphPad Software Inc., San Diego, CA, USA).

## Results

There were in total of 799 A and B dispatch category calls during the study period. In 102 (12.8 %) of these delivery took place before reaching hospital. According to the national birth registry, there were in total of 34 194 births in Helsinki during the study period of which 103 were out-of-hospital. The incidence of EMS managed out-of-hospital delivery was found to be 3.0/1000 births. All pregnancies in Group 1 were single pregnancies. In Group 2 there were 10 twin pregnancies and one triplet pregnancy. The study population is described in Table 1. Statistically significant differences were found in gravidity, parity and gestational age.

**Table 1:** Study population, dispatching statistics and haemodynamics

	Group 1 (*n* = 102)	Group 2 (*n* = 697)	*p*
Age, years (mean, 95 % CI)	31.4 (30.3–32.4)	30.4 (29.9–30.8)	ns
Gestational age, weeks (mean, 95 % CI)	39.6 (39.3–40.0)	37.0 (36.6–37.4)	<0.0001
Gravidity, n (median, IQR)	3 (2–4)	2 (1–4)	0.0071
Parity, n (median, IQR)	1 (1–2)	1 (0–2)	<0.0001
Nulliparous, % (mean, 95 % CI)	11.8 (5.45–18.1)	39.6 (36.0–43.2)	<0.0001
Multiparous, % (mean, 95 % CI)	40.2 (30.5–49.9)	25.8 (22.6–29.1)	0.0025
Duration from the start of phone call to ambulance dispatch, sec (mean, 95 % CI)	147 (133–162)	190 (175–205)	<0.0001
Duration from dispatch to first unit reaching scene, sec (mean, 95 % CI)	430 (392–468)	449 (435–463)	ns
Native Finnish, % (mean, 95 % CI)	54.9 (45.1–64.7)	43.8 (40.1–47.5)	0.0348
Maternal heart rate > 100 bpm, % (mean, 95 % CI)	23.5 (14.3–32.7)	22.5 (19.2–25.9)	ns
Maternal systolic blood pressure < 100 mmHg, % (mean, 95 % CI)	10.5 (3.9–17.1)	5.4 (3.6–7.2)	ns
Maternal systolic blood pressure < 100 mmHg and heart rate > 100 bpm, % (mean, 95 % CI)	4.7 (0.1–9.3)	1.0 (0.2–1.8)	0.0253

**Fig. 1:**
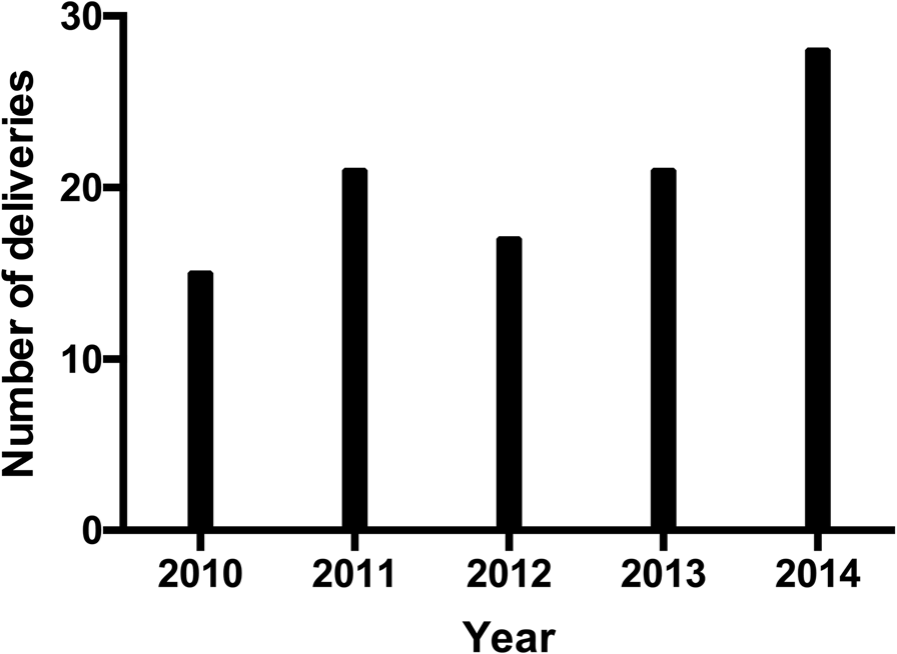
Yearly number of out-of-hospital deliveries

There was an increase in the number of out-of-hospital deliveries during the study period as shown in Fig. 1. Of all the pre-hospital deliveries in 47 (46.0 %) calls the child had already been born before the first EMS unit reached the scene and in 55 (54.0 %) calls the delivery was managed by the EMS staff. MICU physician was present on scene in 76 (74.5 %) of the out-of-hospital deliveries either during the delivery or post-delivery. In the remaining 26 (25.5 %) of the cases the delivery was managed by BLS, ALS or medical supervisor level staff with help of physician teleconsultation.

**Fig. 2:**
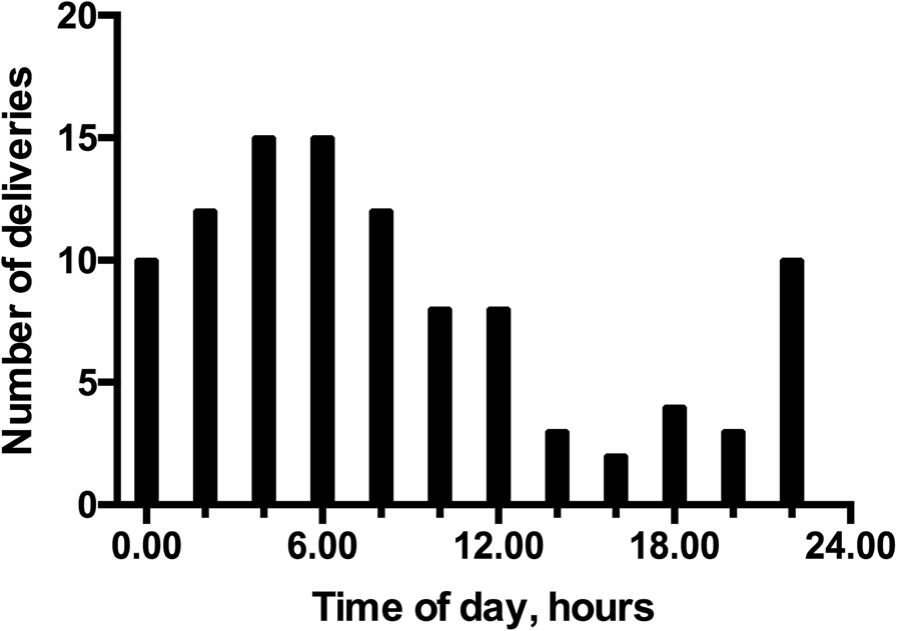
Temporal distribution of out-of-hospital deliveries

There was a statistically significant difference (*p* < 0.0001) between groups when comparing the duration from the beginning of the emergency call to first unit being dispatched as shown in Table 1. Majority of the calls for EMS were made to the patient’s home address, 82.4 % for Group 1 and 84.1 % Group 2. No statistical difference was found between the groups. The temporal distribution of the calls is shown in Fig. 2. The reasons for out-of-hospital delivery determined from the EMS records are shown in Table 2.

**Table 2:** Reasons for out-of-hospital delivery

	Percent
Precipitate birth	70.6
Delivery in an ambulance enroute to hospital	10.8
Delivery in a car or a taxi enroute to hospital	7.8
Planned home birth	6.9
Undiagnosed pregnancy	3.9

The gestational age could be determined from either the ambulance or hospital records for 95 (93.1 %) mothers in Group 1 and 687 (98.6 %) mothers in Group 2. In both groups the mean duration of pregnancies was term as shown in Table 1. In Group 2 four (0.6 %) patients were not pregnant altogether despite the ambulance dispatch being classified as relating to childbirth. In five (4.9 %) of the out-of-hospital deliveries the gestational age was under 37 weeks, the lowest gestational age being 29 + 3 weeks. No stillbirths were detected in our study but there were two spontaneous abortions at gestational ages of 15 + 0 and 21 + 0 weeks, respectively.

Nationality and native language could be determined from the patient records for 681 (85.2 %) mothers in Group 1 and 757 (94.7 %) mothers in Group 2. The whole study population included mothers from 55 different nationalities speaking 42 different native languages. A statistically significant difference (*p* = 0.0349) was found when comparing the proportion of native Finnish people (defined as Finnish nationality and speaking either Finnish or Swedish, the official languages) between the groups as shown in Table 1. The largest ethnic group after Finnish people were Somali with clear over-representation in Group 2 compared to Group 1 (*n* = 110, 15.8 % vs. *n* = 5, 4.9 %, *p* = 0.0035).

### Complications

For out-of-hospital deliveries, no major maternal complications were noted in the study population. No maternal deaths were reported in the patient records. Estimated maternal bleeding was found to be low with a median of 300 ml (IQR 200–400 ml). Single largest bleeding estimate was 1400 ml and bleeding in excess of 1000 ml was reported in only two cases. Third degree perineal tears requiring suturation were reported in four (3.9 %) deliveries. Two (2.0 %) less severe tears requiring suturation (one vaginal, one second degree perineal) were reported.

Means of maternal haemodynamic variables recorded are shown in Table 1. No statistically significant differences were found between groups in either hypotension defined as systolic blood pressure below 100 mmHg or tachycardia defined as heart rate over 100 beats per minute. A statistically significant difference (*p* = 0.0253) between groups was found in combined hypotension and tachycardia as a surrogate for intravascular volume depletion.

No perinatal deaths were reported. For out-of-hospital deliveries Apgar scores for the infants were given by the EMS staff either at one and five minutes after birth if EMS staff was present at birth or immediately after arriving on scene. Mean Apgar scores are shown in Table 3. Mean weight, height and head circumference measured in hospital were within normal ranges as shown in Table 3. One neonate was small for gestational age weighing 2310 g at 39 + 0 weeks of gestational age. One child (1.0 %) required resuscitation and intubation on scene and was admitted to the paediatric intensive care unit and treated for meconium aspiration. The child was discharged from hospital with no observed long-term sequelae.

**Table 3:** Infant statistics

Weight, g (mean, 95 % CI)	3398 (3288–3508)
Height, cm (mean, 95 % CI)	49.5 (49.1–49.9)
Head circumference, cm (mean, 95 % CI)	34.4 (34.1–34.7)
Male sex, % (mean, 95 % CI)	56.4 (46.2–66.7)
Apgar score at 1 min, n (mean, 95 % CI)	8.7 (8.2–9.2)
Apgar score at 5 min, n (mean, 95 % CI)	9.6 (9.3–9.9)
Apgar score on arrival to scene^a^, n (mean, 95 % CI)	9.7 (9.4–10.0)

^a^Birth before first EMS unit on scene, Apgar score given when first unit arrived on scene

## Discussion

The incidence of EMS managed out-of-hospital births in Helsinki was found to be 3.0/1000 births and the amount of out-of-hospital deliveries managed by EMS yearly increased from 15 to 28 during the study period. The incidence is slightly higher than previous studies in Finnish population [2, 6, 7]. As the study was limited to an urban population, this cannot be explained by long travel distances and is more likely to be related to prolonged admission to delivery unit as discussed in a similar study [8]. The amount of births registered by the national birth registry and ambulance records were almost identical thus suggesting a good compliance to national registry reporting and that EMS was involved in practically all of the out-of-hospital deliveries during the study period. Despite the increase in incidence, category A and B calls relating to childbirth still represent a very small minority, around 0.3 %, of all ambulance calls of Helsinki EMS.

Finland has been quite secluded and the population homogenous up until 1990s. This has slowly started to change due to immigration, but by the end of 2014 foreign nationals still represent less than 4 % of the national population. Even so, most of the immigrant population is living in the capital area well represented by the large variance in the nationalities and native languages of the mothers detected in the study. We initially hypothesised that the increase in out-of-hospital deliveries would be due to the increase in immigrant population, especially from Somalia, where a large family size is common as immigrants have been identified as a risk group for out-of-hospital delivery [9]. This seemed to be affecting only the number of ambulance dispatches where no delivery took place out-of-hospital. This may be explained by a language barrier between the caller and dispatcher leading to a lower threshold of alerting EMS when the situation on scene remained unclear during the emergency call.

Even though the incidence of out-of-hospital deliveries increased, amongst the 102 out-of-hospital deliveries no maternal or infant mortality was noted in our study. Great majority of the children born were full term and within normal weight and height. We did not encounter any extreme prematurity as previously described [10, 11], although in our study we considered all births below 22 + 0 weeks of gestational age as spontaneous abortions and these were not included in the data analysis as perinatal deaths as this is the current practice in Finland.

No major complications to either the mothers or the infants causing long-term sequelae directly attributable to the out-of-hospital delivery were detected. This is in contradiction with previous studies, but could be due to the short response times and transfer distances in an urban area. Also the availability of an EMS physician in the MICU could be a factor in managing the births appropriately especially when difficulties arise. The incidence of third degree tears was in the range recently published [12–15] for in-hospital deliveries. Amount of maternal blood loss was found to be low although currently the estimates for blood loss for the out-of-hospital phase are based solely on EMT/paramedic or EMS physician visual estimation and are likely to be inaccurate as suggested by previous studies [16–18].

It is also likely that the small sample size affects the ability of the current study to detect maternal or neonatal morbidity and mortality, as these events have been relatively rare in previous Scandinavian studies [1, 6, 7] despite increasing incidence. Especially premature out-of-hospital births have been linked with increased neonatal morbidity and mortality in a number of studies [10, 11, 19–21]. As noted above, out-of-hospital deliveries present a very small minority of all EMS calls and the ones involving prematurity are an extremely rare occurrence. This causes significant challenges for prehospital staff at all levels in maintaining sufficient proficiency in managing out-of-hospital deliveries when complications arise.

As a clear limitation of the study concerning the infants was the lack of data on body temperature on arrival to hospital, which has previously been linked to increased neonatal mortality [19]. It is possible that during transfer to hospital hypothermia could develop, but if this is evaluated on arrival, it is not systematically entered in the hospital records. In future, body temperature of the child should be recorded in prehospital phase and on arrival to hospital and recording it in the patient records should be added to EMS and hospital protocol. This said no complications attributable to hypothermia could be detected. As the study was performed in a single urban area in Finland it’s generalisability to other countries or rural areas is clearly limited.

## Conclusions

In conclusion, the current study showed an increase in incidence of out-of-hospital deliveries managed by EMS over the five-year study period in Helsinki area. There were no maternal or perinatal mortality or major complications resulting in long-term sequelae associated with the EMS-managed out-of-hospital births.

## Abbreviations

ALS, advanced life support; BLS, basic life support; EMS, emergency medical services; EMT, emergency medical technician; MICU, mobile intensive care unit
